# A Universal Model of Cation Effects in Electrocatalysis

**DOI:** 10.1021/jacsau.5c01115

**Published:** 2025-11-13

**Authors:** Joaquin Resasco

**Affiliations:** McKetta Department of Chemical Engineering, 12330 University of Texas at Austin, Austin, Texas 78712, United States

**Keywords:** heterogeneous catalysis, electrocatalysis, electrochemistry, electrolyte effects, cation effects, CO_2_ reduction

## Abstract

Electrolyte cations are conventionally viewed as inert spectators in electrocatalysis. However, a wealth of observations show that catalytic rates are often highly sensitive to cation identity. Despite their prevalence, these cation effects have resisted a unified mechanistic explanation, with different physical phenomena implicated across reaction chemistries, catalyst compositions, and choice of solvent. In this perspective, we describe a general framework for understanding cation effects in electrocatalysis based on electrostatics. We argue that cations influence reaction rates by modifying the strength of the electric field present at the catalyst surface, which alters the energetics of adsorbed intermediates and transition states according to their dipole moments and polarizabilities. The magnitude of this field depends on how cations arrange at the electrode surface, controlled by their size, shape, solvation, and packing efficiency. Cations that can arrange more densely result in a steeper potential drop at the electrode surface and consequently a stronger electric field. Our model further identifies two criteria for observing cation effects: (1) the operating potential must be negative of the electrode’s potential of zero total charge, ensuring that cations accumulate at the interface, and (2) the energetics of the kinetically relevant elementary step must be field sensitive. This framework reconciles previously inconsistent trends, including why cation effects appear only for some catalysts, why reaction selectivity is sensitive to cation identity, and why activity can increase with cation size on certain metals but decrease on others. Supported by kinetic measurements, spectroscopy, and atomistic simulations, the model provides both conceptual value for building intuition about catalysis at charged interfaces and predictive value for anticipating trends for new reactions, catalysts, and electrolytes. We conclude by highlighting the importance of electric fields across electrochemical, thermochemical, and biological catalysis and propose that considering the electrostatic environment around active sites offers new opportunities for improving activity and selectivity.

## Graphical abstract

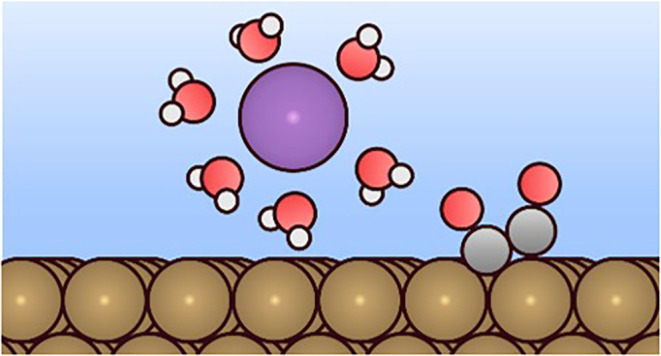

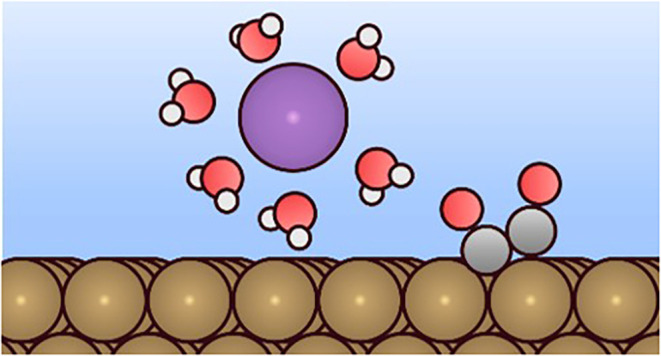

## 1 Introduction

Electrochemical cells typically use mobile ions to conduct charge between spatially separated electrodes. In liquid-phase systems, this ionic conduction generally occurs through the motion of dissolved cations and anions from a supporting electrolyte. The specific identity of these electrolyte ions is conventionally assumed to be inconsequential to the chemistry occurring at the electrode surfaces. However, compelling evidence accumulated over decades reveals that this picture is inaccurate, and that the choice of electrolyte ion can significantly influence catalytic rates.^1–17^

The sensitivity of electrocatalytic reaction kinetics to electrolyte cation identity has been documented for nearly a century.^1^ Since then, these cation effects have been observed across diverse electrocatalytic chemistries, including the hydrogen evolution reaction (HER), the reduction of CO_2_ (CO_2_R), oxygen reduction (ORR), hydrogen oxidation (HOR), nitrate reduction, and the oxidation of small organic molecules.^8–17^

Despite their prevalence, cation effects are not universally observed. Whether and how electrolyte cation choice influences rates depends on the reaction under investigation and catalyst composition. For instance, increasing alkali metal cation size enhances reaction rates for the alkaline HER on noble metal surfaces (Au, Ag, and Cu), but decreases rates over more reactive catalysts (Pt, Pd, and Ir).^10,18^ Similarly, ORR kinetics on Pt display marked sensitivity to alkali cation identity, while rates over Pd surfaces are insensitive to changes in cation size.^13,19,20^ This inconsistent behavior has hindered the development of a unified mechanistic understanding of cation effects in electrocatalysis, and the reasons behind these effects remain the subject of ongoing debate.^21–23^

Many physical arguments have been put forward to describe how cation identity influences electrocatalytic rates.^21^ Unfortunately, most studies related to cation effects limit their descriptions to a single reaction and catalyst composition. This results in descriptions that may be consistent with a limited set of observations, but that entirely fail to explain other well-known behavior. For instance, two of the more commonly invoked mechanisms involve direct noncovalent interactions between cations and specific adsorbates, or cation-induced restructuring of water at the catalyst surface.^21–23^ The former model struggles to account for the ubiquity of cation effects across reactions that involve very different intermediates. If interaction of the cation with a specific adsorbate, for example adsorbed hydroxide or carbon dioxide, were uniquely important, it is difficult to rationalize why cation effects would be observed in reactions where these intermediates are not involved or kinetically relevant. Furthermore, cation effects are also observed for alkylated ammonium and phosphonium-based cations for which direct Lewis acid–base type interactions invoked for alkali metal cations are unlikely to be present.^24–27^ Similarly, the water restructuring model fails to explain the observation that cation effects persist in organic solvents where water is not present and with organic cations that minimally affect solvent structure.^27–29^ This suggests that changes in water structures are not uniquely responsible for observed cation sensitivity in electrocatalysis. Thus, a general model that satisfactorily explains the breadth of cation effects across electrocatalysis is still lacking.

In this perspective, we present such a general model for understanding cation effects in electrocatalysis. Where previous accounts have offered reaction- or adsorbate-specific rationalizations, our framework identifies the unifying electrostatic origin that governs them all. Our model posits that cation identity modifies the strength of the electric field experienced by intermediates and transition states adsorbed to the catalyst surface. Changes in electric field strength modify the energies of adsorbates based on their dipole moments and polarizability, thus reshaping the energetic landscape of the reaction and ultimately altering reaction rates.

The electric field is sensitive to cation identity because the size and shape of cations influences how densely ions can arrange at the electrode surface and how quickly the potential drops at the catalytic interface into solution. Our model suggests two criteria for cation identity to significantly affect rates: (1) the reaction operating potential must result in negative excess charge on the surface, such that cations are electrostatically driven to the interface, and (2) the energetics of the kinetically relevant step must be field sensitive. This framework clearly explains the full diversity of known experimental observations, including the absence of cation effects for certain catalysts, the diverging activity trends with cation size for different metals, and the effects of cation size on selectivity for complex reactions.

This electrostatic model, which is supported by a combination of experimental kinetic and spectroscopic data, as well as atomistic simulations, provides a universal way of understanding electrolyte effects in electrocatalysis. Beyond deepening our fundamental understanding of how the structure of electrochemical interfaces influences surface chemistry, these insights highlight opportunities to improve catalytic performance through rational electrolyte design.

## 2 Overview of This Perspective

In this perspective, we present a general formulation of our electrostatic model along with illustrative examples of how these effects manifest for specific electrocatalytic reactions. Unlike previous reviews that have treated cation effects across different electrocatalytic reactions as isolated phenomena, we will attempt to describe their common mechanistic origins across reactions and catalyst compositions.

We limit our discussion to the influence of cation identity on the intrinsic kinetics of electrocatalytic surface reactions. While cations may influence properties such as reactant and product mass transport,^30^ changes in catalyst structure under operating conditions,^31^ or bulk electrolyte properties,^32,33^ we do not focus on those aspects here. We note that the studies discussed here examined cation effects under conditions in which mass transport limitations were explicitly excluded such that trends were driven by changes in reaction kinetics.

The perspective is structured as follows. We begin by discussing how the strength of electric fields at catalyst surfaces influences reaction rates. This is followed by an analysis of why electrolyte cation identity influences electric field strength at electrochemical interfaces. We then describe the conditions under which cation effects are observed, highlighting two key criteria. The first criterion is that the operating potential of the reaction must be negative of the potential of zero total charge of the electrode. The second criterion requires that the key intermediates and transition states in kinetically relevant elementary reaction steps must be field sensitive. Throughout this discussion, we demonstrate how these variables result in observed trends in reactivity for several important electrochemical reactions.

Our discussion continues with a comparison of this electrostatic model to other descriptions of cation effects proposed in the literature, and shows how our framework resolves previously contradictory observations. We also describe the simplifications and limitations of our electrostatic model.

The perspective concludes with an outlook describing how these insights are generally applicable across electrochemical and thermochemical catalysis and what opportunities these insights afford for improving the activity and selectivity of catalysts through deliberate manipulation of interfacial electric fields.

## 3 Influence of Electric Field Strength on Reaction Energetics

Since our model proposes that cations influence reactivity by modifying the electric field experienced by adsorbates, we begin by examining how electric fields fundamentally affect catalytic reaction rates. We note that a significant body of work exists studying the influence of electric fields on surface chemistry at metal single crystals in ultrahigh vacuum conditions.^34,35^

The effect of an electric field on surface reactivity can be understood using a relatively simple model. The energies of intermediates and transition states adsorbed on a catalyst surface change in response to an electric field depending on their dipole moment and polarizability. This relationship can be expressed mathematically as

$$E=E_{0}+\mu \cdot E-\frac{\alpha E^{2}}{2}+\cdot \cdot \cdot$$ 1

where *E* describes the potential energy of the adsorbed molecule, *E*_0_ is its adsorption energy in the absence of an electric field, μ is its static dipole moment, α is its polarizability, and *E* is the electric field strength. Higher-order terms can generally be neglected for the field strengths relevant to electrocatalysis.^36^ The dipole moment μ and electric field *E* are vector quantities, while the energy *E* is a scalar. The energy of the adsorbate is dependent on both the magnitude and orientation of the field, with this orientation dependence dictated primarily by the geometry of the adsorbate, as the field direction is always perpendicular to the catalyst surface.

To illustrate how this relationship affects reaction rates, consider a simple transformation of adsorbed species A to adsorbed species B on a catalyst surface. As depicted in Figure 1, if the dipole moments or polarizabilities of these intermediates differ, their energies will respond differently to changes in electric field strength. For instance, if species A and B have different dipole moments, the reaction energy for converting A to B on the surface is directly modified by changes in field strength. Thus, by altering the interfacial field, the energetic landscape of the reaction and consequently catalytic rates can be systematically controlled.

**Figure 1:**
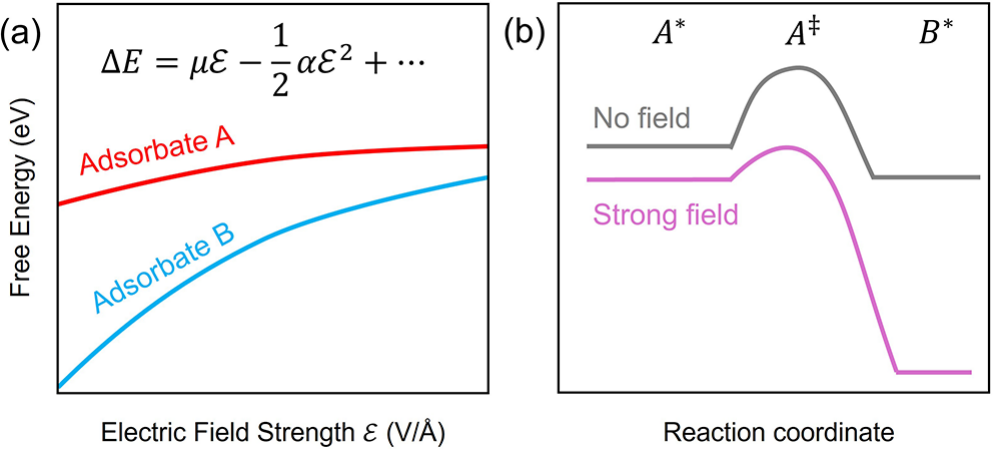
How electric field strength influences reaction energetics. (a) Free energy of an adsorbed species as a function of interfacial electric field strength. The field direction is oriented perpendicular to the surface and becomes increasingly negative from the right to the left side of the plot. This reflects operation negative of the PZTC. (b) Differences in dipole moments or polarizabilities between adsorbed intermediates cause reaction energetics to vary with field strength.

In our example, we have considered changes in the thermodynamic reaction energy arising from differences in dipole moments between intermediates A and B. These changes in initial and final state energies are what have typically been calculated in prior studies.^11,37–39^ As rates depend on activation energies rather than reaction energies, this analysis assumes a Bro̷nsted-Evans–Polanyi type relationship exists between the activation energy and reaction energy. While recent work shows this is likely, more sophisticated calculations could estimate the dipole moment and polarizability of the transition state itself to more accurately describe how reaction kinetics would respond to field variations.^40^ We also note that while our example emphasized changes in dipole moment, field influences on reaction rates can also be driven by changes in adsorbate polarizability.^41^

The impact of electric field strength becomes particularly significant for reactions in which adsorption or desorption steps are kinetically relevant. Since molecules in the adsorbed state experience field stabilization while those in the fluid phase do not, substantial differences in reaction and activation energies emerge as a result of the field for these elementary steps. In our illustrative example, if the adsorption of species A was rate-determining, the overall reaction rate would increase dramatically with increasing field strength due to the stabilization of adsorbed A, and a decrease in its barrier for adsorption.

This scenario is commonly encountered in electrocatalysis. For the CO_2_ reduction reaction, over weakly binding metals such as Au and Ag, the rate-determining step is CO_2_ adsorption.^42–44^ Upon interaction with the electrode surface, CO_2_ accepts electron density, changing its geometry to a bent configuration.^43,45^ It consequently develops a significant dipole moment. This causes the adsorbed state to be strongly stabilized in the presence of a negative electric field relative to fluid-phase CO_2_ in the electrolyte (Figure 2a).^37^ Consequently, the energetics of CO_2_ adsorption, and by extension CO_2_R to CO, become significantly more favorable in the presence of a strong electric field (Figure 2b).^37^ This prediction is supported by both density functional theory (DFT) calculations and experimental measurements of activation barriers for CO_2_R to CO with changing cation size over Ag surfaces (Figure 2b).^27,37^

**Figure 2:**
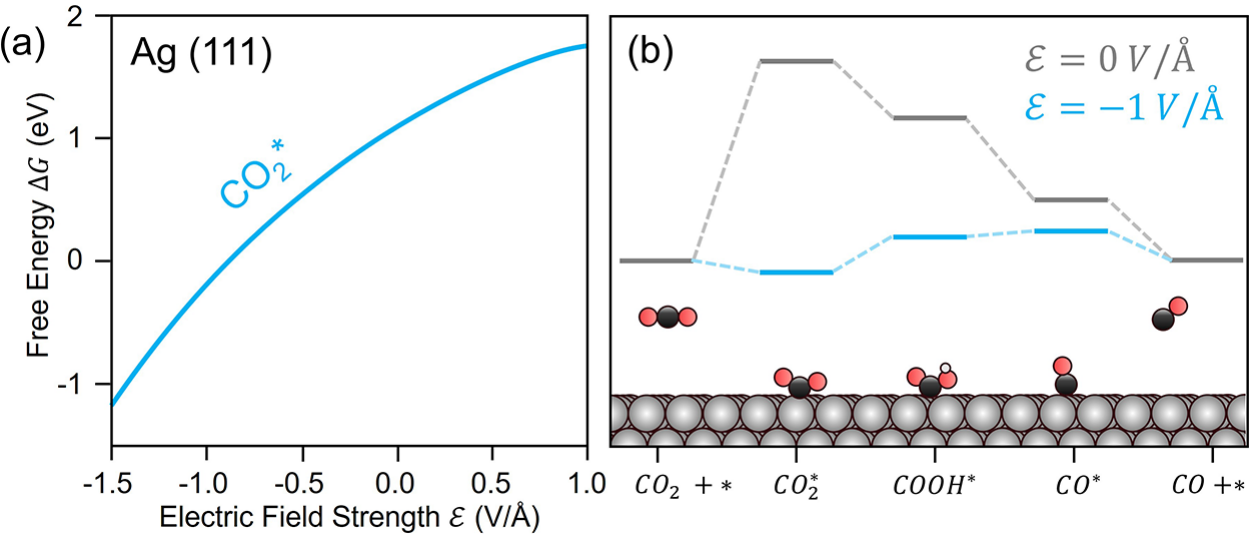
Field effects on CO_2_ reduction. (a) Adsorbed CO_2_ is significantly field-stabilized relative to its precursor in the fluid phase. (b) Reaction coordinate diagram for CO_2_R over Ag (111) at no field and in the presence of a field. As CO_2_ activation is rate limiting over Ag, increasing field strength facilitates CO_2_ reduction and CO formation. Adapted with permission from prior work.^37^ Copyright 2016 American Chemical Society.

A similar effect occurs for the alkaline HER over metal surfaces. For noble metals, such as Au and Ag, the rate-determining step is the Volmer step, which involves hydrogen adsorption onto the surface via water dissociation.^46–48^ A more negative field facilitates this elementary step, thereby increasing reaction rates.^10^ Conversely, for more reactive metals, where the desorption step becomes more difficult, this effect is reversed.^10,49^ This highlights the importance of understanding which elementary steps govern rates over different catalyst materials and understanding how the energetics of these elementary steps respond to changes in the reaction environment.

Having established how electric field strength influences reactivity, in the following section we describe how the choice of electrolyte cation influences field strength at the electrode–electrolyte interface.

## 4 Why Cation Identity Impacts Electric Field Strength

The strength of the electric field at the surface of an electrode depends primarily on the spatial variation of the electrostatic potential. Neglecting heterogeneities across the electrode surface, the field strength is determined predominantly by the distance over which the potential drops from the electrode surface to the bulk electrolyte. A more compact electrochemical double layer results in a steeper potential drop from the electrode to the solution and consequently generates a stronger electric field.

Cation size can modify the thickness of the double layer in two ways. First, it can influence the ion-electrode distance, with larger cations potentially positioned farther from the electrode surface. Second, and more critically, cation size affects the efficiency with which cations can pack in two dimensions at the electrode surface. Larger cations cannot pack as densely, meaning less charge can be contained within a fixed distance from the electrode surface.

We have recently provided experimental evidence supporting this mechanistic picture by studying how the size of quaternary ammonium cations influences CO_2_ reduction in nonaqueous media.^27^ On Ag electrodes, CO formation rates were found to increase monotonically as cation size decreased, with activity following the order tetrahexylammonium (THA) < tetrabutylammonium (TBA) < tetrapropylammonium (TPA) < tetraethylammonium (TEA) (Figure 3a). Direct experimental measurements of interfacial fields using vibrational Stark effect spectroscopy confirmed that smaller cations generate stronger fields, as indicated by higher Stark tuning rates (Figure 3b). This trend has been consistently observed both in aqueous and nonaqueous electrolytes.^25,50–52^ These observations are consistent with the idea that ions with a smaller interfacial radius pack more efficiently at the electrode surface, producing a thinner double layer, a sharper potential drop, and consequently a stronger local electric field (Figure 3c). Complementary kinetic and computational studies further demonstrated that this stronger field accelerates CO_2_ reduction by facilitating the activation of CO_2_ at the interface.^27^

**Figure 3:**
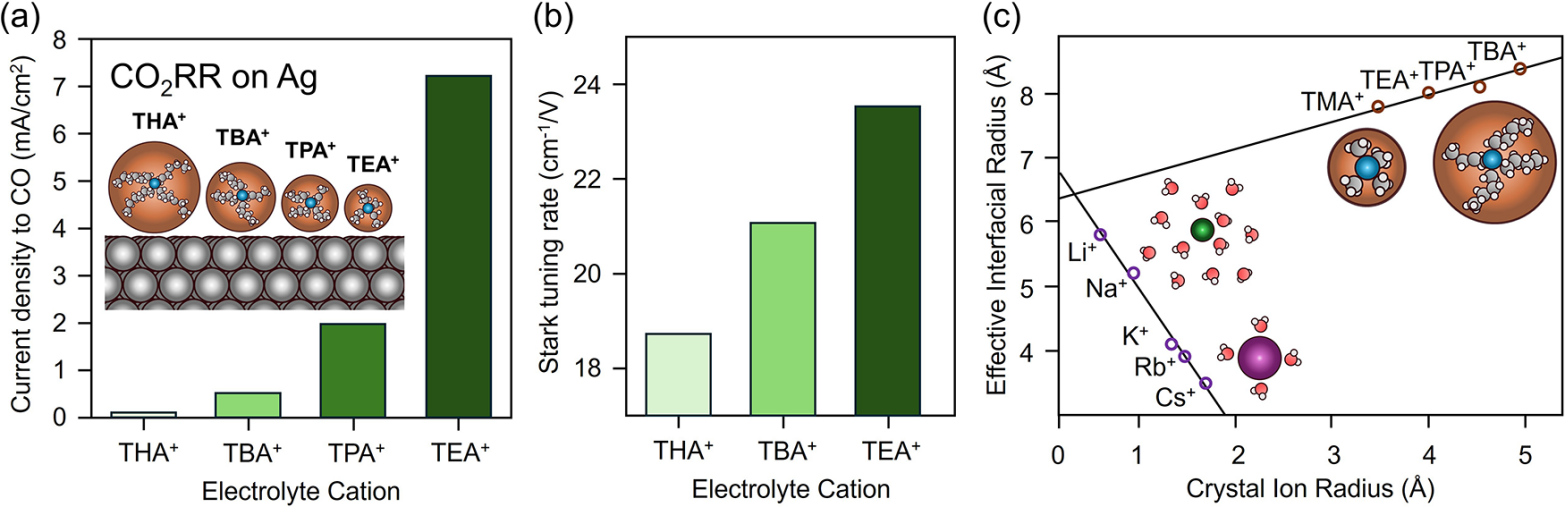
How cation identity influences electric field strength. (a) For organic cations, decreasing cation size increases rates of nonaqueous CO_2_R to CO over Ag catalysts. (b) This increase in rate is due to an increase in field strength, quantified by a larger vibrational Stark tuning rate. (c) For organic cations, increasing the crystal radius decreases surface charge density and weakens field strength. For alkali cations in water, the effective interfacial radius varies inversely with the crystal radius. Adapted with permission from prior work.^12,27^ Copyright 2025 Springer-Nature, 2019 Royal Society of Chemistry.

This conceptual framework can be extended to other cation classes and solvents, provided that solvation is carefully considered (Figure 3c). For aqueous alkali cations, hydration energies vary strongly across the group.^53^ Lithium, despite its small bare ionic radius, exhibits the largest hydrated radius due to its high charge density and strong solvation shell.^54^ In contrast, cesium interacts only weakly with water and thus has the smallest hydrated radius.^54^ The effective ordering of hydrated size therefore inverts relative to the crystal ionic radii, producing a general trend in which higher hydration energies (Li^+^ > Na^+^ > K^+^ > Rb^+^ > Cs^+^) correspond to larger interfacial radii, poorer packing, and weaker fields.^12,18,55^ Vibrational Stark effect studies have directly confirmed this trend experimentally.^56^ In prior studies, we and others also demonstrated that large, weakly solvated cations like Cs^+^ are able to displace more strongly solvated Li^+^ ions at electrocatalytic interfaces, highlighting the key role of solvation in determining which alkali metal cations dominate the interfacial region.^11,56^

For other electrolyte cations, such as quaternary ammonium ions, solvent interactions are considerably weaker and less dependent on cation size.^57^ In these cases, the crystal ionic radius provides a more instructive metric when considering cation behavior at the electrode surface. Computational studies have confirmed this effect, demonstrating that interfacial ionic radii increase with crystal ionic size for quaternary ammonium cations but decrease with ionic radius for alkali metal cations.^12^

As mentioned previously, it is plausible that cation size modifies field strength either by altering the ion-electrode separation distance or by changing cation packing density at the electrode surface. For symmetric ions like alkali metal cations, disentangling these effects proves challenging. However, organic cations, with their highly tunable molecular geometries, allow these effects to be interrogated independently.

We recently made use of geminal phosphonium dication based electrolytes to address this question systematically.^58^ These dicationic species feature two phosphorus cations, each bound to three alkyl chains. A fourth alkyl group tethers the two *P* ^+^ ions to each other.^59^ The length of each alkyl chain can be independently modified synthetically. By maintaining fixed alkyl groups appended to each cation while varying the chain length linking the two cationic centers, we could selectively modify the packing density of cations at the electrode surface (Figure 4a). We anticipated that longer linker groups would reduce the density at which cations could arrange at the surface—a hypothesis that we subsequently confirmed using classical molecular dynamics simulations and electrochemical impedance spectroscopy (Figure 4a).^58^ Comparing dications with different linker lengths, we demonstrated that cation packing density at the electrode surface significantly influences reactivity for CO_2_R to CO, with higher packing density generating stronger fields that facilitate CO_2_ adsorption and CO formation (Figure 4b).^58^

**Figure 4:**
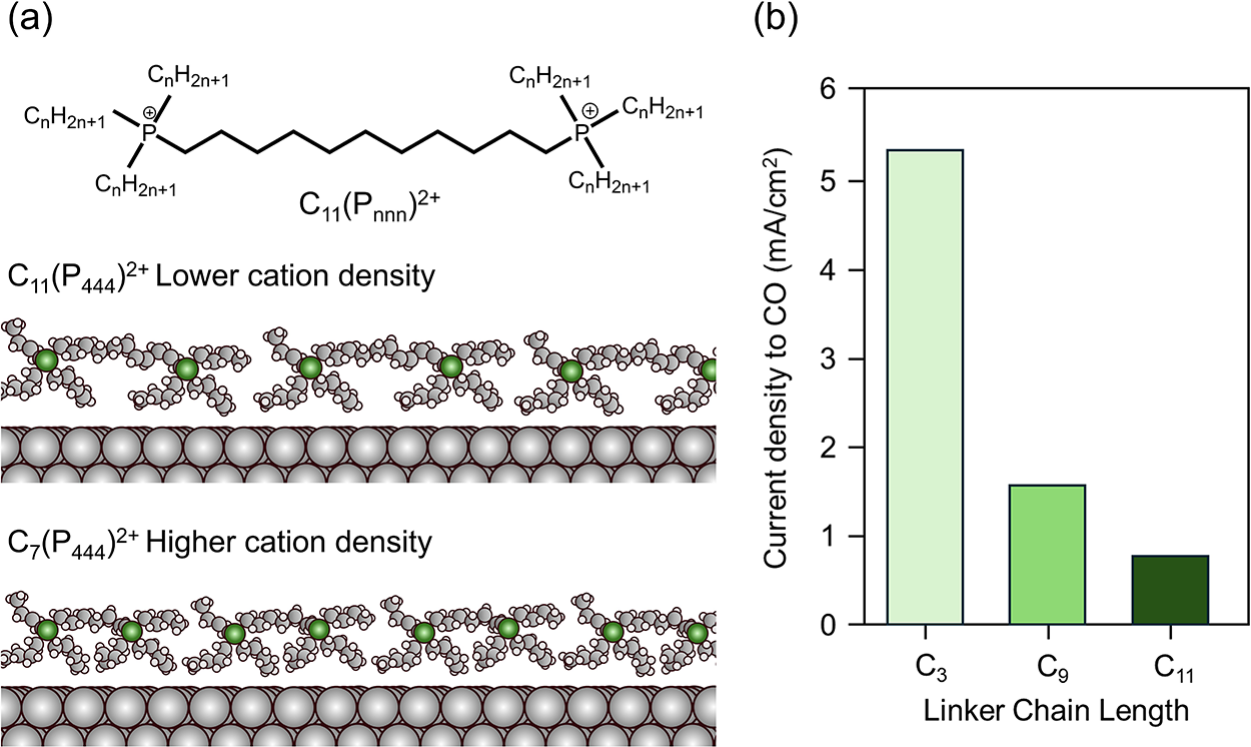
Interfacial cation concentration influences catalytic rates. (a) Phosphonium dications allow systematic tuning of surface cation density by tuning the linker chain length. (b) Decreasing cation density at the catalyst surface by increasing linker length inhibits rates. Adapted with permission from prior work.^58^

These tailored dications also enabled us to independently evaluate the importance of cation-electrode distance. By fixing the linker length at a large value while systematically modifying the lengths of the other substituent groups, we could isolate the effect of changing cation-electrode distance at constant packing density. These experiments also revealed a notable effect, with shorter cation-electrode distances leading to increased reaction rates.^58^

Beyond influencing activity for simple reactions governed by adsorption steps, these electrostatic considerations also rationalize more complex phenomena, such as the cation-dependent selectivity observed in CO_2_R on Cu surfaces. Prior studies have shown that increasing cation size systematically enhances the Faradaic efficiency toward multicarbon products, while lowering selectivity to H_2_ and C_1_ products (Figure 5a).^11,12,60^ Through kinetic and intermediate-feeding studies, we previously identified that the two steps most strongly influenced by cation size in CO_2_R are CO_2_ activation and C–C coupling.^11^ The same electrostatic arguments applied above explain these observations: stronger interfacial fields stabilize CO_2_ on the electrode surface relative to molecular CO_2_, thereby promoting activation, and C–C coupled intermediates are more strongly stabilized than their uncoupled precursors.^11^ CO dimerization is generally considered the key pathway to multicarbon products over Cu surfaces,^61–63^ and the resulting adsorbed CO–CO intermediate is more field-stabilized than the separated adsorbed CO reactants (Figure 5b).^11^ Thus, increasing the interfacial field strength from Li^+^ to Cs^+^ lowers the barrier to C–C coupling and accelerates multicarbon production. We also showed that this argument holds for other coupling pathways, such as the reaction between adsorbed CO and CHO, where the coupled product is more field sensitive than its precursors.^11^ Beyond the C–C coupling step, it is possible that C_2+_ adsorbates have varying field sensitivity. Changes in field strength may therefore influence the selectivity among multicarbon products (e.g., ethylene vs ethanol). However, these effects have not been thoroughly investigated.

**Figure 5:**
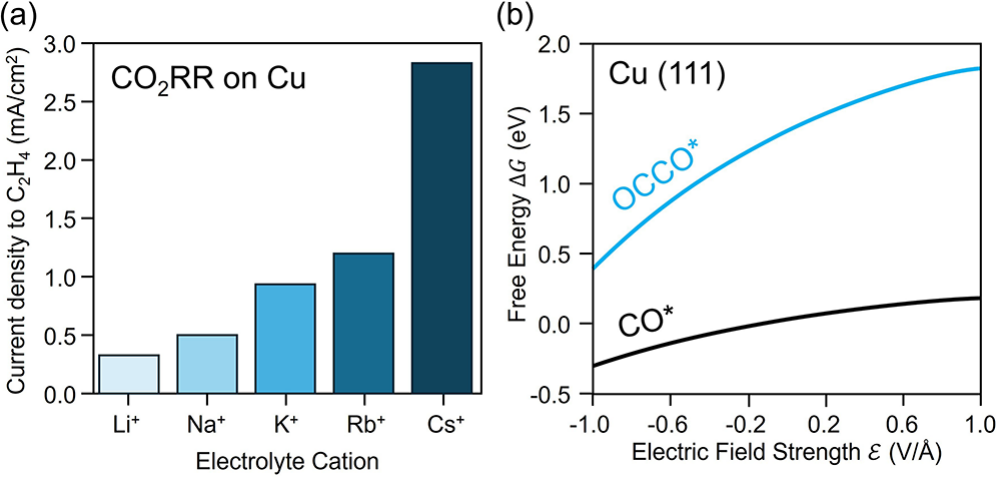
Cation effects on reaction selectivity. (a) For CO_2_R over Cu, increasing cation size systematically increases kinetic rates of formation of C_2_H_4_ and other multicarbon products. (b) Density functional theory calculations show that this is due to field stabilization of C–C coupled intermediates relative to their precursors. Adapted with permission from prior work.^11^ Copyright 2017 American Chemical Society.

These results demonstrate that cation-controlled fields not only dictate activity for simple reactions but also direct selectivity in complex multielectron transformations such as CO_2_R on Cu.

Collectively, these findings establish that cation identity modifies interfacial electric field strength through two primary mechanisms: by altering the distance between the ionic charge centers and the electrode surface, and by changing the packing density of cations at the interface. Both factors contribute to determining the steepness of the potential drop across the electrochemical double layer and consequently the magnitude of the electric field experienced by adsorbed species. This connection between cation properties and electric field strength provides the mechanistic link needed to understand how electrolyte composition influences electrocatalytic performance.

## 5 When Cation Identity Impacts Rates

Having established that electrolyte cation identity influences catalytic activity by modifying the electric field experienced by reaction intermediates, we now turn to the critical question of predicting when cation effects will be observed in electrocatalytic systems. Although cation effects are widespread in electrocatalysis, there are notable instances where cation identity does not influence reaction rates, or where its effects on rates diverge depending on catalyst composition.

We propose that there are two fundamental requirements for cation identity to have an important influence on rates in electrocatalysis. First, the operating potential of the reaction must be negative of the potential of zero total charge (PZTC) of the catalyst. Second, the energetics of the adsorbates involved in kinetically relevant reaction elementary steps must be sensitive to electric field variations. We employ the PZTC as a descriptor rather than the more commonly referenced potential of zero charge (PZC) or potential of zero free charge (PZFC). While the PZC defines the potential at which the surface carries no excess charge without adsorbates, the PZTC accounts for the influence of adsorbed species, which can be present at significant coverages during electrocatalytic reactions of interest.^64,65^ PZC or PZTC values can be measured experimentally using electrochemical charging or impedance techniques and estimated computationally.^20,66–68^ Measuring these values can be complicated by potential-dependent changes in adsorbate or solvent coverage;^69,70^ nevertheless, surface charge remains a conceptually and, in many cases, predictively useful descriptor of cation effects.^20,71^

At potentials negative of the PZTC, the electrode carries excess negative charge, and oppositely charged cations are electrostatically driven toward the catalyst surface (Figure 6a). Once at the interface, these cations modify the electric field experienced by adsorbed species. This field modulation only influences catalytic activity if the energetics of the kinetically relevant steps are field sensitive. Conversely, at potentials positive of the PZTC, cations are repelled from the interface, rendering surface chemistry largely insensitive to cation identity (Figure 6b).

**Figure 6:**
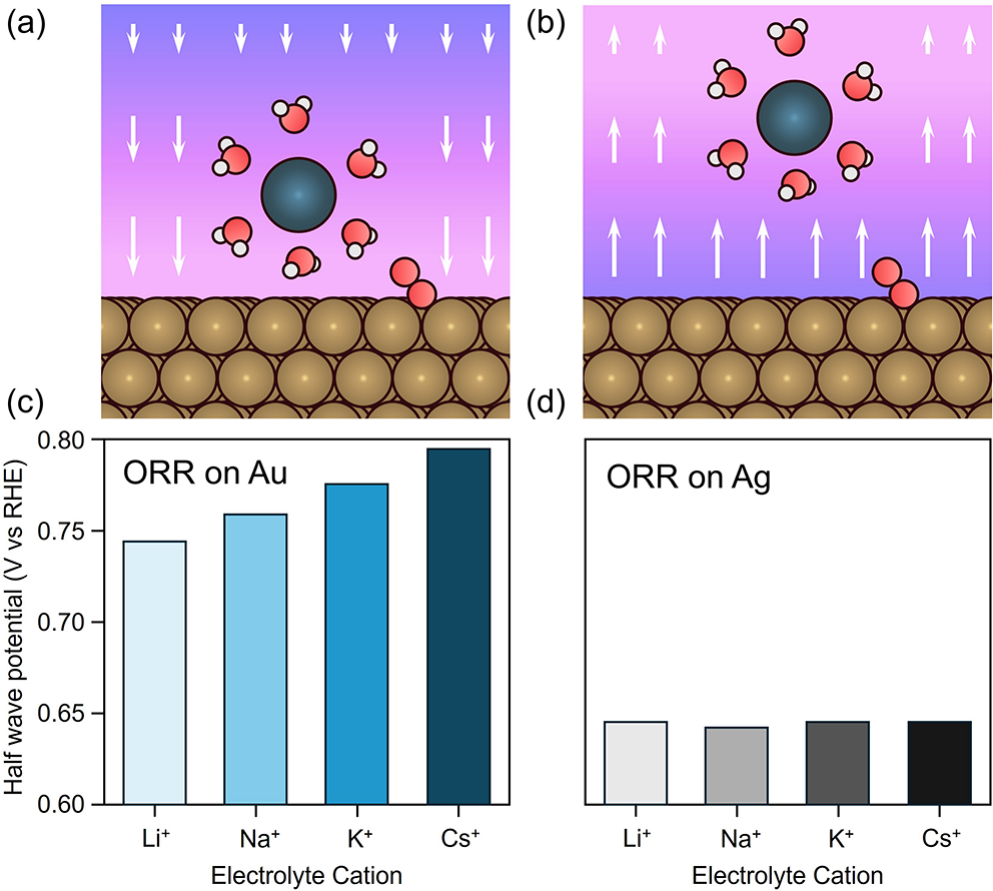
Catalyst surface charge predicts the presence of cation effects. (a) At operating potentials negative of the potential of zero total charge of the metal cations are driven to the catalyst surface. (b) At operating potentials positive of the PZTC, cations are electrostatically repelled from the surface. (c) Thus, cation identity affects rates for ORR over metals like Au, whose PZTC is positive of the ORR operating potential, but not Ag (d) whose PZTC is negative of the ORR region. Adapted with permission from prior work.^20^ Copyright 2024 American Chemical Society.

We recently investigated cation effects on the oxygen reduction reaction across a range of transition metal surfaces to determine whether metal surface charge could predict these effects across different catalysts.^20^ Consistent with our predictions, metals with operating reaction potentials negative of their PZTC exhibited pronounced cation effects, while those with a PZTC more negative than the ORR potential did not. For instance, despite Pt and Pd having quite similar interaction energies with adsorbates, rates over Pt increase progressively from Li^+^ to Cs^+^ containing electrolytes, while over Pd, rates remain largely insensitive to cation identity.^20^ A similar effect was observed for Ag and Au (Figure 6c,d).^20^

Our kinetic analysis revealed that cation effects originate from changes to O_2_ activation energetics for noble metals and from hydroxide removal energetics for more reactive metals. Apparent activation energies decreased with increasing alkali cation size, consistent with negative fields facilitating O_2_ adsorption.^20^ Reaction order analysis suggested that increasing cation size slightly destabilized adsorbed OH.^20^

Having demonstrated that surface charge effectively predicts cation effects for the ORR, we sought to generalize this concept across diverse electrocatalytic reactions.^71^ We selected probe reactions with operating potentials spanning a wide voltage range: the HER, ORR, and the oxidation of methanol, ethylene glycol, and glycerol. As shown in Figure 7, the onset potentials for these reactions over Pt span a broad range relative to the PZC of various metals. Here the PZC is used for simplicity due to the difficulty in estimating coverages across this wide range of reactions.

**Figure 7:**
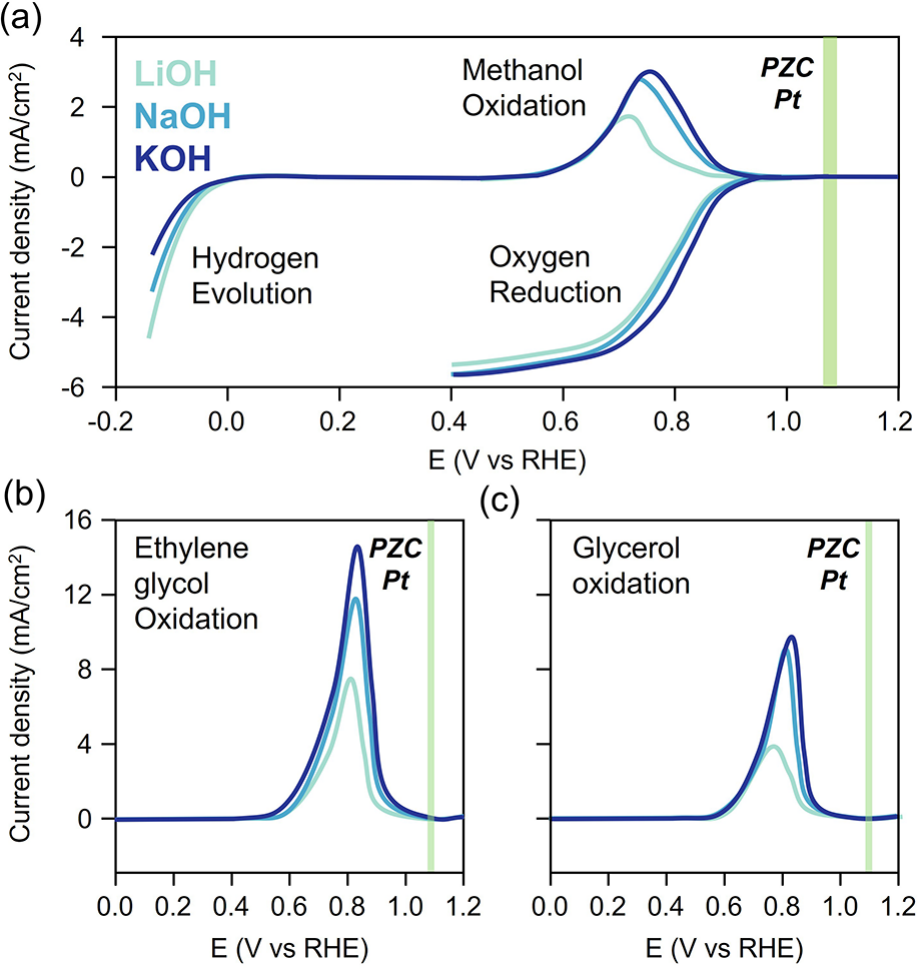
Generalizing surface charge as a descriptor of the presence or absence of cation effects. Cation effects are observed over Pt for reactions which occur negative of its PZC. (a) HER rates decrease with cation size, while ORR and methanol oxidation rates increase with cation size. PZC values are taken from prior work.^67,73^ (b), (c) Ethylene glycol and glycerol oxidation rates increase with cation size. Reproduced with permission from prior work.^71^

We studied each reaction in alkaline conditions with alkali metal hydroxide electrolytes on Pt, Pd, Au, and Ag catalysts.^71^ Pt and Pd have similar adsorption properties but different PZC values, allowing us to decouple these effects.^72^ Similarly, Au and Ag both bind adsorbates weakly but the PZC of Ag is far more negative than that of Au.^72^ According to our framework, we expected to observe cation effects when the applied potential was negative of the PZC of the metal.^71^ Consistent with this prediction, Pt showed cation effects for all reactions studied (Figure 7). Pd exhibited effects for the HER, and oxidation of methanol, ethylene glycol, and glycerol oxidation, but not for the ORR. Cation effects were observed over Au for the HER and ORR but for none of the oxidation reactions, which occurred at higher potentials, positive of the PZC. Finally, Ag, with its very negative PZC, only showed cation effects for the HER. These results provide compelling evidence that surface charge effectively and generally predicts when cations will accumulate at electrocatalytic surfaces and influence electric field strength.

To demonstrate the second criterion for observing cation effects, namely that the energetics of kinetically relevant steps must be field sensitive, we recently examined the HER across various transition metals.^10^ The HER occurs at potentials negative of the PZC for essentially all transition metals, regardless of pH. In acidic media, the reaction proceeds via an initial proton-coupled electron transfer step to form adsorbed hydrogen. This is denoted the Volmer step. The Volmer step is followed by either a Langmuir–Hinshelwood type reaction between two adsorbed hydrogen atoms (Tafel step) or a second Eley–Rideal type PCET step (Heyrovsky step) to form the product H_2_. Independent of the mechanism, adsorbed hydrogen is the sole reaction intermediate, and thus catalyst activity correlates with the free energy of adsorption of hydrogen.^46,47^

We hypothesized that since adsorbed hydrogen lacks a significant dipole moment on metal surfaces, it would have minimal field sensitivity, rendering HER rates largely insensitive to electrolyte cation identity in acidic media. We confirmed this experimentally by measuring HER rates as a function of cation size across metals with diverse hydrogen binding energies.^10^ No influence of electrolyte cation identity was observed over Pt, Pd, Ir, Cu, Ag, or Au. Ab initio molecular dynamics simulations verified that cations do accumulate at the electrode interface under these conditions. This implies that cation presence alone is insufficient to modify reaction rates, and that field sensitivity is necessary for observing cation effects.

In contrast to acidic conditions, under alkaline conditions, water molecules serve as the hydrogen source through deprotonation at the catalyst surface. We hypothesized that the transition state and products of water dissociation would be significantly field sensitive, resulting in the observation of cation effects in base. Consistent with this expectation, we observed pronounced cation effects across all metals studied in alkaline media. Noble metals, whose rates are limited by water dissociation, showed enhanced activity in the presence of larger cations due to increased interfacial field strength, while more reactive metals displayed inverse trends. This resulted from excessive field-induced intermediate stabilization, which impedes product desorption.^10^

These findings establish that to observe cation effects in electrocatalysis, two criteria must be satisfied. First, the operating potential of the reaction must be negative of the catalyst’s PZTC to ensure cation accumulation at the interface, and second, the energetics of the kinetically relevant step must be field sensitive. These requirements explain the seemingly inconsistent manifestation of cation effects across different electrocatalytic systems and provides a predictive framework for anticipating when electrolyte composition will significantly influence catalytic performance.

While our discussion has focused on negatively charged surfaces where cations accumulate, analogous arguments could potentially be extended to describe effects of anion identity. At potentials positive of the PZTC, anions become the dominant species in the double layer and could, in principle, produce analogous anion effects. However, in contrast to cations, many anions specifically adsorb on metal surfaces, introducing additional complexities that can mask purely electrostatic influences.^6^ As a result, trends with anion identity may largely reflect differences in site blocking or adsorption energetics rather than systematic field effects. Exploring these distinctions represents an interesting direction for future work.

## 6 Compatibility with Other Models

It is important to situate our electrostatic framework in context with other models that have been proposed in the literature. A wide range of mechanistic models have been proposed to explain cation effects in electrocatalysis.^21–23^ These models have clear value, as each captures experimentally observable phenomena and has deepened our understanding of electrolyte–catalyst interactions. However, they often fall short in providing a general framework that explains the full breadth of observations across reactions, electrolytes, and solvents. Rather than treating models of cation effects as mutually exclusive, we suggest that many of these models may represent different ways of describing the same underlying electrostatic phenomena, each emphasizing different aspects of the interface.

One widely discussed framework is the water structure model. In this picture, cations are viewed as kosmotropes (structure-making ions) or chaotropes (structure-breaking ions) that modulate the hydrogen-bond network of interfacial water.^53,74,75^ There is strong evidence that cations alter bulk water structure in ways that depend on their hydration enthalpies and effective radii.^53,74,75^ More recently, vibrational spectroscopic studies have directly demonstrated ion-specific restructuring of water at charged interfaces.^8,76–82^ These studies show that alkali metal cations and organic cations produce distinct changes in the orientation and bonding of interfacial water molecules, which in turn correlate with shifts in reaction kinetics. Such results provide clear evidence that cations influence water organization at catalytic interfaces.

The water structure model can also be reframed in electrostatic terms. Cations influence the orientation and organization of interfacial water dipoles, which in turn modulates the local electric field experienced by adsorbates. In this view, water structure effects may not be entirely distinct from electrostatics, but rather a manifestation of how cations reshape the interfacial field by reorienting solvent dipoles.

Nonetheless, there are important limitations to this water-centric view. Cation effects are routinely observed in nonaqueous solvents where water is absent,^27–29^ and also among organic cations in both aqueous and aprotic media, where solvation enthalpies are smaller and vary only weakly with ion size.^57^ Such findings suggest that cation effects cannot be attributed solely to water structuring. In any polar solvent, the local electrostatic environment is jointly determined by cations and solvent molecules. The extent of solvent reorganization and field screening will depend on the dielectric strength and polarizability of the solvent, such that highly polar solvents may partially attenuate cation-induced field variations. However, experiments show that trends with cation identity are remarkably consistent across aqueous and nonaqueous electrolytes^27^ and that changes in Stark tuning rates with cation size are nearly independent of solvent dielectric properties.^50^ These observations indicate that while solvent polarization modulates the interfacial field, it does not qualitatively alter the electrostatic origin of cation effects.

While interfacial spectroscopy clearly demonstrates that solvent orientation depends on cation identity, it remains far less clear how these structural changes influence reaction energetics. Establishing such connections is challenging: density functional theory cannot adequately sample solvent structures, ab initio molecular dynamics yields trajectories too short for statistical convergence, and classical molecular dynamics lacks the accuracy to describe reaction energetics.^83,84^ Consequently, most studies observe ion-specific solvent structuring but fail to clearly establish the connection to catalytic rates. Solvent reorganization effects can also be interpreted within Marcus-type or coupled ion–electron transfer frameworks, which we view as conceptually compatible with the electrostatic picture advanced here, but these often fail to connect parameters to specific reaction elementary steps.^85,86^

Another common explanation invokes direct interactions between cations and specific adsorbates. For instance, adsorbed OH has been proposed as the key intermediate leading to observed cation effects in the HER/HOR, ORR, and the methanol oxidation reaction over Pt-group metals,^13,16,87^ while adsorbed CO_2_ is often implicated in CO_2_R.^88^ These proposals have intuitive appeal and are supported by spectroscopic correlations. However, even for these reactions the explanation breaks down: pronounced cation effects are observed on catalysts where OH coverage is negligible,^20^ and for CO_2_R on Cu, multicarbon selectivity remains strongly cation-dependent even though CO_2_ activation is not kinetically relevant to their formation.^89^ In fact, identical cation effects are seen for the reduction of CO_2_ and CO over Cu, clearly indicating that a specific cation–CO_2_ interaction cannot be the determining factor.^11,82,90^ Moreover, there are a wealth of electrocatalytic transformations which exhibit strong cation effects despite not involving these intermediates at all.^91,92^

From a broader perspective, many of these specific interaction models may also be reinterpreted in electrostatic terms. Noncovalent interactions, such as hydrogen bonding or ion–dipole stabilization, are fundamentally manifestations of electrostatics.^93^ What we call hydrogen bonds in chemical language can be represented as the electrostatic interaction of point charges and dipoles (Figure 8). The distinction lies largely in conceptual framing rather than underlying physics. Thus, cation–adsorbate and cation–solvent interactions can be unified within an electrostatic framework that emphasizes how local fields alter adsorbate energetics, regardless of whether we describe the effect in primarily chemical or physical terms. Recent studies have highlighted interfacial electric fields as central to catalytic reactivity, aligning with the view advanced here.^52,88,94,95^ Together, these perspectives suggest that the field is increasingly recognizing electrostatics as a common thread underlying many observed cation effects.

**Figure 8:**
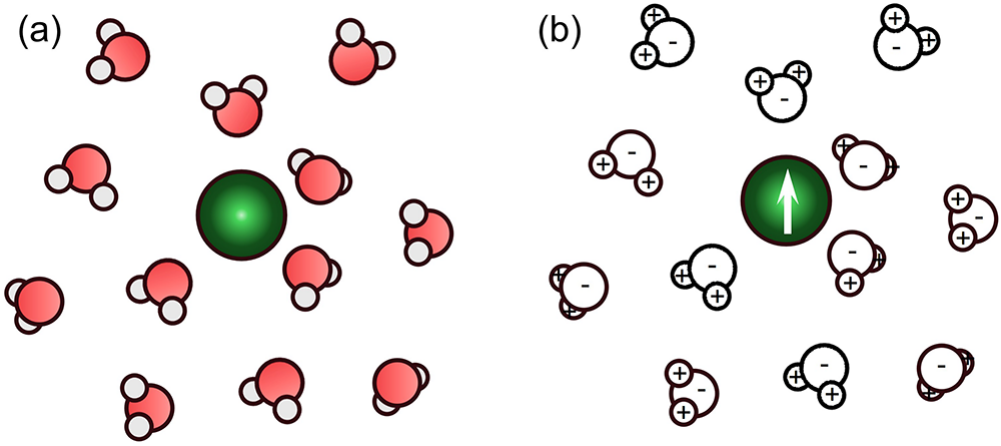
Chemical and electrostatic views of noncovalent interactions. (a) In the chemical picture, interactions are described as hydrogen bonding between water molecules. (b) In the electrostatic picture, the same interaction is represented as point charges and dipoles interacting through Coulombic relationships. Adapted with permission from prior work.^96^ Copyright 2015 American Chemical Society.

## 7 Limitations of the Electrostatic Model

As with any simplified description of complex interfacial phenomena, our electrostatic framework cannot capture every molecular detail. In the spirit of the adage that “all models are wrong, but some are useful”, we view this as the simplest model capable of explaining the breadth of experimental observations regarding cation effects in electrocatalysis while retaining predictive power. While we recognize that electric field strength is not the only property of the electrochemical interface that changes with cation size, nor that it offers a complete description of the complex structure of electrochemical interfaces, we believe this model offers both (1) conceptual value, by building intuitive connections between interfacial structure and reactivity, and (2) predictive value, by capturing broad trends across diverse reactions, catalysts, and electrolytes.

There are several simplifying assumptions that underlie this model. First, we have implicitly assumed that the potential of zero total charge is independent of cation identity. In our description, field strength is determined by the arrangement of cations at the interface, rather than by shifts in the PZTC relative to the operating potential. Preliminary experimental work suggests that the potential of zero charge can indeed vary with cation identity.^18,55^ While no clear systematic trend or mechanistic explanation for this variation has yet emerged, future work should explore whether PZTC shifts contribute meaningfully to cation effects.

Second, we have treated the electrode–electrolyte interface as a mean-field system, neglecting spatial heterogeneity in the local electric field. Atomistic simulations indicate that field strength can vary substantially as a function of distance from individual cations.^37,47^ Nonetheless, when the near-surface cation concentration is high, as both experimental and computational studies consistently show for negatively charged interfaces,^18,55,95,97^ overlapping field distributions will converge to yield an effective, spatially averaged field that closely approximates mean-field behavior. Furthermore, our own dication experiments suggest that interfacial cation density must be substantial under catalytic operating conditions. Otherwise, variations in linker length would likely not produce measurable effects on catalytic rates.

Finally, while the electrostatic model provides a consistent qualitative framework for understanding cation effects, achieving quantitative predictive accuracy remains an important open challenge. Current models typically assume static solvent configurations and average field strengths. Moreover, linking these thermodynamic relationships to actual kinetics requires knowledge of how transition-state energies respond to the interfacial field—an area where little data currently exist.^40^ Future progress toward quantitative prediction will likely require integrating dynamic solvent sampling, site-resolved field characterization, and calculation of field dependent transition-state energies.

We note that qualitative cation trends are robust across catalyst morphologies. Single crystals, polycrystalline foils, and nanoparticle catalysts consistently exhibit the same trends with cation size, and computations on low-index facets recover the same behavior.^10,11,13,20,49,60,90,98^ While magnitudes of cation effects can vary with surface structure,^90,99^ any putative field enhancement from curvature or edge sites should only matter when the feature size approaches the length scale over which the interfacial potential drops. For roughness on larger length scales, the interface is essentially planar from the perspective of an adsorbate, and the field it experiences is essentially unchanged compared to a bulk material.

Resolving these open questions—whether local fields are heterogeneous, how water (or other solvent) structures vary around each cation, and how dynamic these interfacial structures are—will require the development and application of new tools. More accurate atomistic simulations capable of accessing relevant time and length scales, as well as advanced spectroscopies such as two-dimensional infrared (2D-IR) or ultrafast techniques, could provide useful insights.^84,100^ At the same time, we believe that reactions themselves can often serve as the most sensitive probes of interfacial properties, since even small perturbations to local electric fields or ion distributions can produce measurable changes in rates, selectivity, or kinetic signatures. Thus, the design of clever kinetic experiments—using probe reactions, intermediate-feeding studies, isotopic labeling, or detailed reaction order analysis—will be a powerful route for testing and refining new physical models. Similarly, electrochemical impedance spectroscopy provides a powerful and experimentally accessible means of probing interfacial structure.

## 8 Importance of Electric Fields across Heterogeneous Catalysis

In this perspective, we have described how the choice of electrolyte cation influences the electric field experienced by adsorbates and how this in turn influences catalytic rates. However, we believe thinking about the role of the electrostatic environment surrounding active sites, and how it can be controlled, is generally useful across different areas of catalysis. While different mechanisms may be available for modifying electric field strength around the active site, the model described here can still be used to understand the consequences for reactivity across diverse catalytic systems. Here, we descibe some examples of how electric fields can influence reactivity across different areas of catalysis.

In electrocatalysis, the electric field strength is dependent on pH as well as cation identity.^101^ Most energy conversion reactions of interest involve transfer of protons and electrons. The equilibrium potentials of these reactions are pH independent of the reversible hydrogen electrode (RHE) scale.^102^ However, surface charge responds primarily to the absolute electrode potential relative to the potential of zero charge of the catalyst under study (e.g., the standard hydrogen electrode). The result is that at a fixed overpotential, or applied potential versus RHE, increasing electrolyte pH makes the absolute potential more negative compared to the potential of zero charge of the surface. In alkaline conditions, the electric field is therefore much more negative.^103^

This has important consequences on reactivity. We recently demonstrated that this field argument can be used to rationalize pH effects on the oxygen reduction reaction.^104^ Metals like Au and Ag are very poor catalysts in acidic conditions but their activity increases significantly with increasing pH.^105–107^ Conversely, metals like Pt show limited pH dependence.^108,109^ In our study, we showed that changes in electric field strength can explain systematic trends observed in ORR rates over metal catalysts with pH. Through detailed kinetic studies, we identified how the energetics of kinetically relevant steps of the ORR change moving from acidic to alkaline conditions, and how these changes can be rationalized by an electrostatic model.^104^ Recent computational studies have also attributed changes in ORR reactivity with pH to changes in electric field strength.^38,68^

In thermochemical catalysis, field effects can also be important. Promoter effects in thermochemical catalysis have long been rationalized through an analogous electrostatic framework, where alkali metal promoters modify local electric fields at catalyst surfaces and thereby tune adsorption energies and reaction barriers.^34–36,41,110^ In solid–liquid systems, despite there being no externally applied potential, a spontaneous electric field is generated due to a mismatch in the Fermi level of the metal and the solution potential.^111^ To equilibrate this difference, there is charge exchange across the interface, with accumulation of electrons or holes at the surface of the metal and accumulation of oppositely charged ions on the solution side. Recent studies have experimentally confirmed the spontaneous generation of this field and shown that it has important consequences on reactivity.^111–115^

Finally, in biological systems, nature’s catalysts, enzymes, represent the most sophisticated demonstration of electric field-mediated catalysis. Their remarkable ability to catalyze difficult reactions stems largely from their capacity to generate precisely oriented electric fields within active sites.^116^ Seminal computational studies have argued that electrostatic preorganization is fundamental to enzymatic catalysis, creating environments that preferentially stabilize transition states over reactants, thereby lowering activation barriers.^117,118^ These theoretical insights have been validated by experimental work showing that enzymes can generate extraordinarily strong and geometrically precise electric fields at active sites that selectively stabilize charge-separated transition states, enabling their exceptional activity.^119–121^ Recent work has demonstrated that the principle of electrostatic catalysis can even be used in a predictive way to substantially improve the catalytic activity of natural enzymes.^122^ The remarkable activity of enzymes highlights the potentially transformative potential of controlling electric fields in heterogeneous catalysis.

The examples presented here, from cation effects in electrocatalysis to pH-dependent reactivity, spontaneous polarization in thermochemical systems, and enzymatic catalysis, illustrate that electric fields around active sites play an important role in catalysis across diverse environments. Understanding and controlling these fields offers a powerful way for designing more efficient and selective catalysts. As the field advances, we anticipate that electric field engineering will become an increasingly important tool for developing more efficient and selective catalysts across all areas of heterogeneous catalysis. The concepts outlined in this perspective therefore have general importance extending well beyond cation effects in electrocatalysis, providing a foundation for understanding and manipulating reactivity through electrostatic interactions at catalytic interfaces.

## 9 Conclusions and Outlook

In this perspective, we have presented a general electrostatic model for understanding cation effects in electrocatalysis. Our model posits that cation identity modifies the strength of the electric field experienced by intermediates and transition states adsorbed to the catalyst surface, with changes in electric field strength altering the energies of adsorbates based on their dipole moments and polarizability. This electrostatic framework explains how cations reshape the energetic landscape of electrochemical reactions and ultimately influence reaction rates through two key criteria: the reaction must operate at potentials negative of the potential of zero total charge, and the kinetically relevant steps must involve field sensitive intermediates or transition states.

Our electrostatic model offers significant advantages over previous descriptions of cation effects. Unlike models that invoke specific interactions between cations and particular adsorbates or rely exclusively on cation-induced water restructuring, our framework provides a unified explanation for the breadth of experimental observations across diverse reactions, catalyst compositions, and electrolyte conditions. It successfully accounts for puzzling phenomena such as the absence of cation effects on certain catalysts, diverging activity trends with cation size for different metals, and the persistence of cation effects in nonaqueous solvents and with organic cations. The generality of our model stems from its foundation in fundamental electrostatic principles that govern all solid–liquid interfaces, rather than reaction- or catalyst-specific mechanisms.

Beyond activity, our framework also explains why cation identity strongly influences selectivity in complex multielectron transformations. For CO_2_R on Cu, larger, weakly solvated cations promote C–C coupling by preferentially stabilizing transition states involved in C-C bond formation, thereby shifting product distributions toward multicarbon products. These examples illustrate that cation-controlled fields can be leveraged not only to accelerate rates but also to steer selectivity, offering a powerful and underexplored dimension of catalyst design.

The conceptual value of this electrostatic framework extends well beyond cation effects in electrocatalysis. As we have demonstrated, electric field effects represent a unifying principle across diverse catalytic environments, from pH-dependent reactivity in electrochemical systems to spontaneous polarization in thermochemical catalysis and the sophisticated field-mediated catalysis observed in biological enzymes. Understanding and controlling interfacial electric fields therefore offers a powerful approach for catalyst design and optimization across all areas of heterogeneous catalysis.

Continued development of operando spectroscopic techniques will be valuable for directly probing electrochemical interfaces and validating the predictions of our electrostatic model. Advanced spectroscopic methods that can characterize interfacial electric field strength, ion distributions, and adsorbate configurations under operating conditions will provide crucial insights into the molecular-level processes governing catalytic activity. Similarly, more sophisticated computational approaches that explicitly account for solvent structure, constant applied potential, electric field strength, and the presence of ions will be essential for quantitative predictions and rational catalyst design.

However, we believe there is significant power in much simpler kinetic measurements. Reaction rates are often the most sensitive probe of local conditions at catalytic interfaces, providing information that is extremely difficult to obtain through physicochemical characterization or computational techniques alone. Cleverly chosen probe reactions or probe molecules can reveal clear information about interfacial electric fields, ion distributions, and adsorbate stability that would otherwise remain hidden. The systematic variation of electrolyte composition, pH, and applied potential in kinetic studies provides a particularly powerful approach for testing mechanistic hypotheses and extracting fundamental insights about electrostatic effects in catalysis. We therefore anticipate that kinetic studies will continue to have an important and central role in advancing our understanding of electric field effects in heterogeneous catalysis.

Beyond deepening our fundamental understanding of catalysis at solid–liquid interfaces, the insights presented here offer practical routes to improve catalytic activity and selectivity. Recognizing that electrolyte composition can actively manipulate interfacial electric fields opens new opportunities for rational catalyst optimization through electrolyte design. Rather than treating ions as inert spectators, practitioners can now strategically choose ions and solvents to tune the electrostatic environment, favor desired pathways, or suppress competing reactions. This electrolyte engineering approach complements traditional strategies focused on catalyst composition and structure. The same principles extend to deliberate design of catalytic materials that exploit interfacial field effects. The PZC or PZTC of a catalyst can be shifted to influence field effects, for instance through alloying or surface functionalization.^123^ More broadly, jointly optimizing catalyst composition and the surrounding environment to maximize field sensitivity represents a promising direction for enhanced catalytic performance.

The electrostatic framework presented here thus provides both fundamental insights into the molecular origins of cation effects and practical opportunities for advancing electrocatalytic technologies. As our understanding of electric field effects in catalysis continues to evolve, we anticipate that electrostatic considerations will become an increasingly important component of catalyst design across electrochemical energy conversion, chemical synthesis, and environmental remediation applications.
